# Mating and parenting experiences sculpture mood-modulating effects of oxytocin-MCH signaling

**DOI:** 10.1038/s41598-020-70667-x

**Published:** 2020-08-12

**Authors:** Joseph Phan, Lamees Alhassen, Allan Argelagos, Wedad Alhassen, Benjamin Vachirakorntong, Zitong Lin, Nayna Sanathara, Amal Alachkar

**Affiliations:** 1 Department of Pharmaceutical Sciences, University of California, Irvine, 356A Med Surge II, Irvine, CA 92697-4625 USA; 2 Institute for Genomics and Bioinformatics, School of Information and Computer Sciences, University of California-Irvine, Irvine, CA 92697 USA

**Keywords:** Neuroscience, Medical research, Molecular medicine

## Abstract

The two hypothalamic neuropeptides oxytocin and melanin concentrating hormone (MCH) share several physiological actions such as the control of maternal care, sexual behavior, and emotions. In this study, we uncover the role for the oxytocin-MCH signaling pathway in mood regulation. We identify discrete effects of oxytocin-MCH signaling on depressive behavior and demonstrate that parenting and mating experiences shape these effects. We show that the selective deletion of OXT receptors from MCH neurons increases and decreases depressive behavior in sexually naïve and late postpartum female mice respectively, with no effect on sexually naïve male mice. We demonstrate that both parenting experience and mood-regulating effects of oxytocin-MCH are associated with synaptic plasticity in the reward and fear circuits revealed by the alterations of Arc expressions, which are associated with the depressive behavior. Finally, we uncover the sex-dependent effects of mating on depressive behavior; while the sexual activity reduces the basal levels of depressive behavior in male mice, it reduces in female mice evoked-depression only. We demonstrate that the oxytocin-MCH pathway mediates the effects of sexual activity on depressive behavior. Our data suggest that the oxytocin-MCH pathway can serve as a potential therapeutic target for the treatment of major depression and postpartum mood disorders.

## Introduction

The neuropeptide oxytocin regulates social behaviors such as bonding, mating, and maternal/paternal care^1–8^. Oxytocin levels rise during mating in males and females in animals and humans, and may mediate the anti-depressant and anxiolytic/calming effects of sexual activity^9,10^. Parturition, on the other hand, is associated with a surged release of oxytocin from the paraventricular nucleus (PVN) and supraoptic nucleus (SON), resulting in a rapid initiation of maternal behaviors such as aggression against intruders, positive bond with offspring, postpartum anxiety and depression^11–15^. The hypothalamic neuropeptide melanin concentrating hormone (MCH) shares several physiological actions with oxytocin such as the regulation of emotion, social recognition and interaction, maternal care, sexual behavior, reproduction and stress^15–25^. Genetic deletions of the peptide (MCH-KO) or its receptors (MCHR1-KO) result in mothering deficits similar to those seen in oxytocin receptor KO (OXTR-KO) mice^26^. Both MCH and oxytocin lower the threshold for the initiation of maternal behavior, but are not involved in its maintenance^26,27^. Therefore, the oxytocin and MCH systems may interact to modulate maternal behavior. While only 4% of oxytocin neurons express MCHR1, OXTR mRNA are expressed in the majority of MCH neurons and only rarely in other neurons in the lateral hypothalamus (LH)^28,29^. Electrophysiological studies report that oxytocin interacts and selectively excites MCH neurons but not any other LH neurons^28^. The very selective action of oxytocin and specific location of its receptors on MCH neurons suggests that these neurons may mediate or modulate some of the oxytocin actions on maternal behavior and emotion^28^. We recently showed that the MCH and oxytocin systems interact directly^29^, nonetheless, whether oxytocin regulates maternal- or mating-regulated mood through oxytocin-MCH signaling is unknown. It is plausible to speculate that oxytocin may exert parts of its facilitating actions on mating and maternal responses through interacting with MCH neurons. Consequently, we examined the role of oxytocin-MCH signaling in maternal behavior and mood, and whether this pathway is involved in mating-induced antidepressant effect. For this purpose, we used a Cre/loxP recombinase-technology to selectively delete OXTR from MCH neurons.

## Material and methods

### Animals

#### Generation of MCHCre^OXTRfl/fl^ conditional knockouts

oxtr^flox/flox^ female mice (Oxtrtm1.1Wsy homozygous, Jackson Laboratories, USA) were crossed with male MCH-Cre mice (Tg(Pmch-cre)1Lowl/J (Jackson Laboratories, USA) that contained one transgenic allele expressing Cre recombinase^25,29–31^. The offspring thus had the following genotypes: (1) oxtr^+/flox^, (2) oxtr^+/flox^, Cre. To generate MCHCreOxtr^fl/fl^ KO mice, male oxtr^+/flox^, Cre mice, which have germ cell expression of Cre recombinase were bred with female oxtr^flox/flox^ mice. This led to the following genotypes: (1) oxtr^+/flox^, Cre (2) oxtr^+/flox^, (3) oxtr^flox/flox^, Cre and (4) oxtr^flox/flox^. The third genotype is being the MCH specific-tissue conditional OXTR KO (OXTR-cKO) (Fig. 2a).

#### MCHR1-KO mice

MCHR1-KO mice were generated as previously described^30,32^; MCHR1-KO mice were backcrossed to a BL6-Taconic background for 10 generations, and littermates were then separately bred to generate Wild type (WT) and MCHR1 knockout (MCHR1-KO) mice.

### Subject animals and experimental design

To assess maternal behaviors during the postpartum period, female MCHR1-KO, OXTR-cKO mice and their control littermates were mated with control male mice for 3 days. Following this mating period male mice were removed from the cage and the female mice were subsequently monitored for signs of pregnancy daily by visual examination and weight measurements. The date of birth of pups was considered postpartum day 0 (PPD0). Once pups were born, they were counted and weighed. Maternal behavior observations were carried out in the order of: nest building (PPD1–PPD3), pup weight and milk production (PPD1–PPD21), pup mortality and cannibalism rate, defined as missing pups’ rate (PPD1–PPD5), pup retrieval (PPD1–PPD3), and maternal aggression (PPD7). All mother mice evaluated for the different maternal measures were primiparous.

Maternal behaviors were also assessed in virgin mice, by measuring pups’ retrieval duration and latencies for four consecutive days.

Immobility time in forced swim experiments was used as a measure of the depressive behavior in animals. For this test, naïve 10–12 weeks’ male and female MCHR1-KO, OXTR-cKO and their control littermates were used. Also, the forced swim test was conducted on PPD5, PPD16, PPD21, and PPD30 on a sub-group of the mice that were tested before mating. To assess the effects of sexual activity on depressive behavior, male and female OXTR-cKO and control mice of the same genotype were allowed to mate for 3 days, and were then tested in forced swim test 24 h after the last contact. All experimental procedures and protocols were approved by the Institutional Animal Care and Use Committee of the University of California, Irvine (UCI) and were carried out in consent with the national and institutional standards for the handling and utilization of laboratory mice.

### Behavioral assays

#### Forced swim

The forced swim assay was performed as previously described^33^. Mice were placed individually in a transparent glass cylinder containing water (24 cm high, 14.5 cm diameter, 14 cm water depth) at 23–25 °C. Mice were videotaped for 6 min, and the immobility time (time spent passively floating) was recorded for the last 4 min, after discarding activity in the first 2 min during which an animal tries to escape. ANY-MAZE software was used to record and analyze immobility (Stoelting Co.).

#### Maternal behavior assays in postpartum mothers and in virgin mice

##### Pup mortality and mother’s cannibalism rate

Pup mortality and mother’s cannibalism rate were assessed from PPD1–PPD5. The pups’ mortality was expressed as survival rate for the first 5 days postpartum (PPD1–PPD5) and presented as the percentage of pups that survived in each day compared to the previous day. Cannibalism rates were displayed as total cannibalism after 5 days postpartum.

##### Nest building

Prepartum nesting building behavior was evaluated 3 days after the separation of the pregnant female into a single home cage. In addition, nesting behavior was assessed on PPD1, PPD2, and PPD3 using the 0–5 scale of nest quality^27,34^.

##### Pups’ retrieval assay with postpartum mice

The assay was conducted on PPD1, PPD2, and PPD3, in which, a maximum of five minutes of video recording time to calculate the mother’s latency and duration. The mother was temporarily removed from the cage, and pups were removed except for three pups, which were placed on each of the three corners of the cage (not the nest corner). The mother was then returned to her nest and the latency and duration of pups’ retrieval were recorded.

##### Pups’ retrieval with virgin female mice

Pup retrieval tests were performed on both wild type and knockout female virgins as we described before^30^. All tests were videotaped for a total time of 15 min and analyzed. 24 h before testing time, female virgins were single housed and were provided fresh nesting material. During testing time, the female virgin mouse was temporarily removed from her cage and three of 3-day-old pups were placed in each corner of her cage. The female was returned back to her nest and the latency to retrieve the first pup (in seconds) and the duration of retrieval for the other two pups were recorded for 15 min.

##### Maternal aggression

On PPD7, the aggression behavior assay was utilized to measure the mother’s protectiveness of her pups. The pups were removed from the mother’s home cage, and a stranger male mouse was introduced into the home cage, and the total number of attacks and aggressive actions including aggressive moves, flank/back, head/neck, or combination were measured.

##### Milk production

During PPD1-PPD21, average pups’ weight per mother were measured, and the daily changes in weight were evaluated to calculate the approximation of milk yield from the following equation^35^:

$$ [{\text{Yield }}\;({\text{g/day/pup}}) = 0.0322 + (0.0667 \times {\text{weight}}) + (0.877 \times {\text{gain}})]. $$

Weight: average pups’ weight (g) and gain: (g/day). To obtain reliable and replicable data, milk production results were analyzed for mothers who have only gave birth to 6–8 surviving pups.

### RT-PCR

Quantitative-RT-PCR was carried out using gene-specific primers to mouse Pro-oxytocin (Pro-oxt) and mouse GAPDH. Total RNA was extracted from the whole hypothalamus using phenol/guanidine isothiocyanate (TRIZOL; Invitrogen, Carlsbad, CA), and 2 µg total RNA was reverse transcribed using Superscript III ribonuclease H reverse transcriptase kit (Invitrogen) in the presence of 100 ng random hexamers. Reactions for the quantification of mRNAs by PCRs were carried out using iTag Universal SYBR Green Supermix (Bio Rad), and analyzed by Bio Rad CFX Connect Real-Time System (Bio Rad, CA, USA). The copy number for each target gene and internal control were determined in duplicate assay from each of the standard curve within the exponential range. The expression of genes was calculated relative to that in the virgin female mice and normalized for the GAPDH mRNA, using the 2^∆∆CT^ method^27^.

### Immunohistochemistry (IHC)

Ninety minutes after the forced swim test, mice were anesthetized with halothane and perfused intracardially with saline and 4% paraformaldehyde (PFA). Brains were removed and 20 µm coronal sections were cut using cryostat; three sections were selected from each region of interest according to the mouse brain atlas^36^. Sections were blocked with 4% normal donkey or goat serum in PBS with 0.3% Triton X-100 for 60 min. Brain sections were incubated in the blocking buffer that contains one or mix of the primary antibodies (rabbit anti-Arc 1:500, Sigma, Cat.# SAB4200515, Lot. 098M4794V, chicken Anti- tyrosine hydroxylase (anti-TH) 1:500, Aves, Cat.# F-1005, Lot. TYH73787982). The sections were then washed with PBS, and incubated for one hour with the secondary antibodies (1:500). Sections were washed with PBS, incubated for five minutes with 4′,6-diamidino-2-phenylindole (DAPI) solution (1:10,000), and mounted with Aquamount mounting solution. Image acquisition was carried out using confocal laser microscope. Images were captured using Leica Sp8 TCS confocal microscope (UCI optical biology core facility). TH positive neurons (TH^+^ neurons) and Arc^+^ neurons were counted in the bilateral areas of each section, and the mean values of three non-consecutive sections per brain of 4–6 brains were calculated. In the ventral tegmental area (VTA), the number of Arc^+^ neurons was presented as absolute values and as percentage of the total TH-positive neurons. Brain regions were defined according to their anatomy using Franklin and Paxinos Brain Atlas^36^. For Arc^+^, TH^+^ cell counts, three sections from each brain were stained and selected brain slices, chosen in each animal, according to standard anatomical markers. All cell counts were carried out using ImageJ^37^, and confirmed manually by two persons blind to the experiment conditions.

### Combined fluorescent in-situ hybridization (FISH) and IHC

Double staining of oxytocin receptors and MCH peptide was carried out using fluorescence In-Situ Hybridization via RNA scope and standard IHC as described previously^38^. Twenty-micron brain sections at the level of the lateral hypothalamus were incubated in a quenching solution containing H_2_O_2_ at room temperature for 45 min. Sections were then mounted on Fisherbrand Superfrost Plus Microscope Slides and baked at 60 °C overnight in the hybridization oven. RNAscope in situ hybridization was then carried out using RNAscope Multiplex Fluorescent Kit following the manufacturer’s instructions. The brain sections were then blocked using a blocking buffer that contains 10% normal donkey serum 0.3% Triton X-100 for 1 h at room temperature. The sections were then incubated with anti-PMCH antibody (rabbit polyclonal, antibody courtesy of W. Vale, Salk Institute, La Jolla, CA, USA; diluted 1:375,000) for overnight. The secondary donkey anti-rabbit Alexa 488 antibody (1:250; Jackson Immuno) was utilized to view MCH immunoreactivity. Sections were cover-slipped using RNAscope DAPI, followed by Invitrogen ProLong Gold Antifade-Mountant. Images were captured using Leica Sp8 TCS confocal microscope.

### Statistical analysis

GraphPad Prism (GraphPad Software, Inc.) was used for statistical analysis, and all data were presented as mean ± standard error mean (SEM). *t* test was used to analyze the results of the forced swim tests in MCHR1-KO and WT, nest building, maternal aggression, and survival rate. Pro-oxt mRNA levels in the hypothalamus were analyzed using one-way ANOVA followed by Tukey post-test. The results of forced swim tests, pups’ retrieval and Arc and TH staining were analyzed using two-way ANOVA followed by multiple comparisons test. *P* value < 0.05 was deemed statistically significant.

## Results

### Hypothalamic oxytocin expression changes across pregnancy and postpartum periods

**Figure 1:**
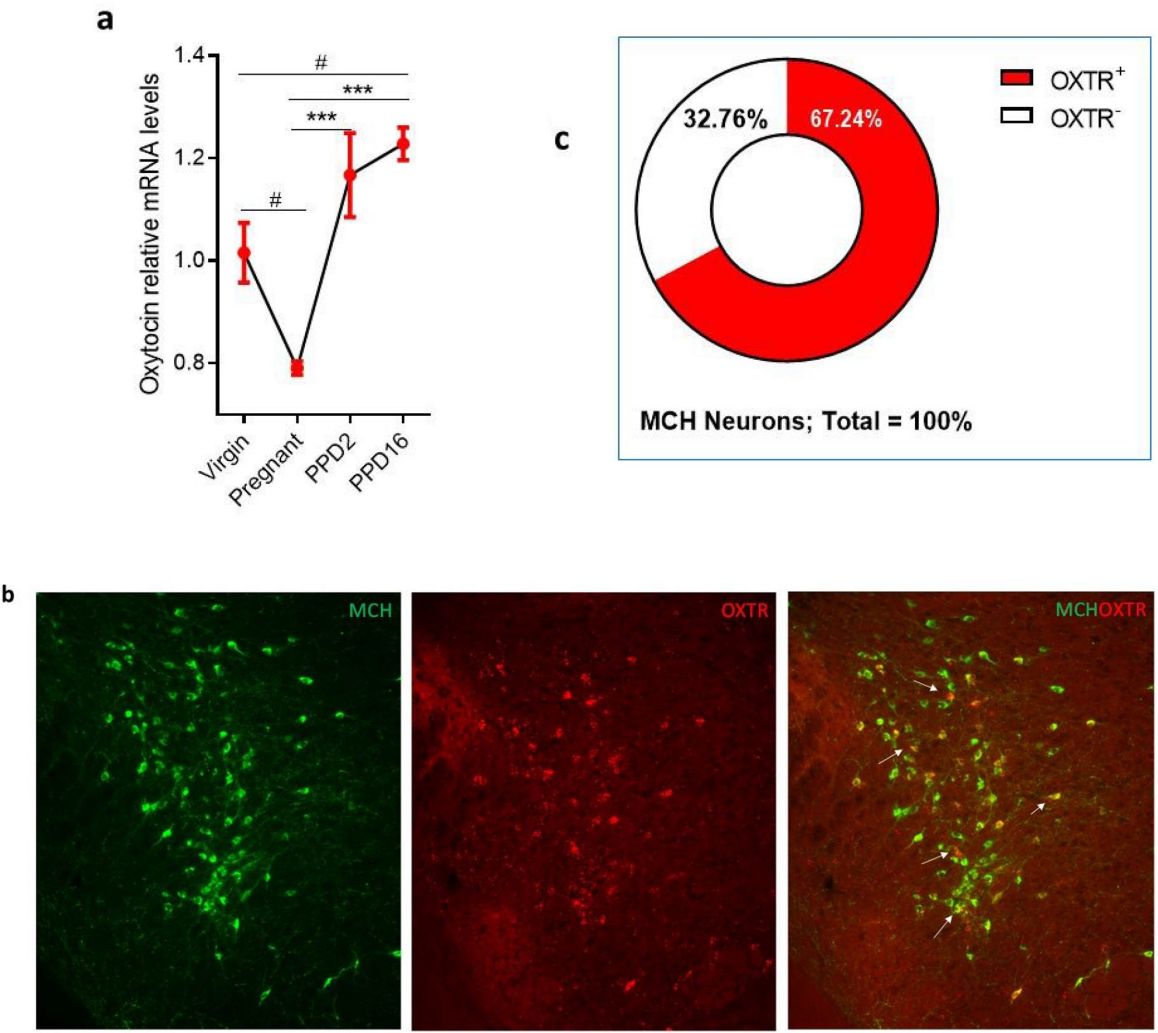
Oxytocin mRNA levels increase in postpartum female mice, and OXTR in the lateral hypothalamus is mainly expressed on the MCH neurons. (**a**) Quantitative real-time PCR of Oxtr mRNA in the hypothalamus in sexually naïve (virgin) and mother mice (early (PPD2) and late (PPD16) postpartum stages), one-way ANOVA followed by Tukey post-test (F_3,23_ = 13, *P* < 0.0001), ^###^*P* < 0.001, ^##^*P* < 0.01 compared to the virgin, ***P < 0.001, compared to the pregnant stage (n = 6 mice/group). (**b**) Representative images of combined FISH and immunostaining showing the co-localization of expression of OXTR mRNA (red) in MCH neurons (green) in the lateral hypothalamus. (**c**) Percentage of MCH neurons that express OXTR mRNA (n = 3).

Pro-oxt mRNA levels in the hypothalamus of pregnant mice were lower than in virgin mice *P* < 0.05 one-way ANOVA followed by Tukey post-test (Fig. 1a). On PPD2 and PPD16, Pro-oxt mRNA significantly increased compared to the pregnant mice, *P* < 0.001, and Pro-oxt mRNA levels on PPD16 were also higher than in virgin mice, *P* < 0.05 one-way ANOVA followed by Tukey post-test. These results indicate that the need for oxytocin increases across the postpartum stage toward the weaning stage.

### OXTR in the lateral hypothalamus is mainly expressed on the MCH neurons

To identify OXTR-expressing neurons in the lateral hypothalamus, RNAScope-FISH method was used. Using double staining of OXTR mRNA (FISH-RNAscope) and MCH peptide (IHC), we found that 67 ± 6% of MCH neurons express OXTR (Fig. 1b,c).

### Deletion of the OXTR from MCH neurons does not affect maternal behaviors in virgin or postpartum mothers

**Figure 2:**
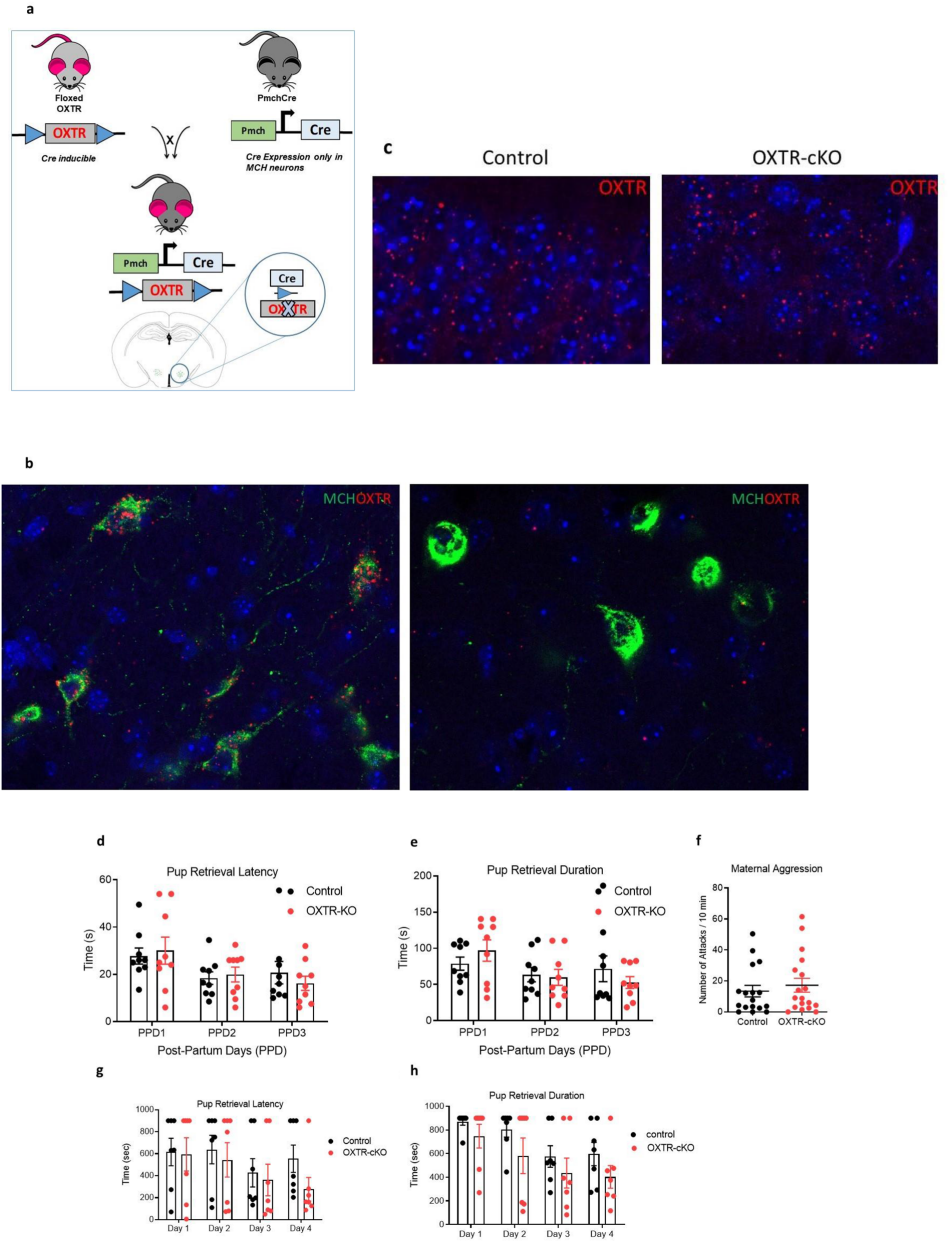
Deletion of the OXTR from MCH neurons does not affect maternal behaviors in virgin or postpartum mothers. (**a**) Schematic representation of the generation of MCHCre^OXTRfl/fl^ conditional knockouts (Brain Image Credit: The Mouse Brain Atlas^36^). (**b**) Representative images of FISH and immunostaining showing the conditional deletion of OXTR mRNA in the MCH neurons of mouse of control and OXTR-cKO mice (counterstained with DAPI, blue). (**c**) FISH staining of the CA1 of the hippocampus showing the intact expression of OXTR in OXTR-cKO. (**e**,**f**) pups’ retrieval in postpartum mice: (**d**) latency to retrieve the first pup, (**e**) duration to retrieve three pups (n_control_ = 9, n_OXTR-cKO_ = 9, *P* > 0.05, two-way ANOVA). (**g**) maternal aggression by postpartum mice (n_control_ = 17, n_OXTR-cKO_ = 18, *P* > 0.05, unpaired *t* test). (**h**,**i**) pups’ retrieval by sexually naïve female mice: (**g**) latency to retrieve the first pup, (**h**) duration to retrieve three pups (n_control_ = 7, n_OXTR-cKO_ = 7, *P* > 0.05, two-way ANOVA).

The selective deletion of OXTR from MCH neurons in the LH was verified using double staining of OXTR mRNA and MCH peptide. Figure 2b illustrates the expressions of OXTR on MCH neurons in the LH in control animals and the OXTR-cKO mice. The expression of OXTR remains intact in other regions of the brain in OXTR-ckO (as shown in the hippocampus, Fig. 2c), demonstrating the selectivity of OXTR deletion from the MCH neurons. Breeding success rate was nearly 100% for both control and OXTR-KO mothers producing an average initial litter size of 6.28 ± 0.48 and 6.33 ± 0.58 respectively (Fig. S1a).

Pups’ mortality was assessed and expressed as a survival rate on the first five postpartum days (PPD1–PPD5). Average control pups’ survival rates were 81.23%, 74.29%, 81.67%, 88.46%, 88.89% onPPD1, PPD2, PPD3, PPD4, and PPD5 respectively. Survival rates of OXTR-cKO pups were: 82.08%, 66.81%, 91.67%, 97%, 100% on PPD1, PPD2, PPD3, PPD4, and PPD5 respectively. Mean differences of the survival rates between the control and OXTR-cKO groups were not significant, *P* > 0.05, two-way ANOVA (Fig. S1b). Cannibalism rates were similar in the control and OXTR-cKO groups, *P* > 0.05, *t* test (Fig. S1c).

Both control and OXTR-cKO mothers displayed high quality nest building behaviors. The average nest building scores were: 4.32 ± 0.18, 4.52 ± 0.19, and 4.57 ± 0.17 on PPD1, PPD2, PPD3 respectively for control mothers, and 4.46 ± 0.6, 4.5 ± 0.13, and 4.48 ± 0.13 on PPD1, PPD2, PPD3 for OXTR-cKO mothers. The mean differences in nest building quality between the two groups in all tested postpartum days were not significant, *P* > 0.05, two-way ANOVA (Fig. S1d).

Since MCH and oxytocin are critically involved in milk production during lactation period, we speculated that the two neuropeptides interact to regulate milk production. However, our results show no significant difference in milk production between the OXTR-cKO and control groups across all measured days, *P* > 0.05, two-way ANOVA followed by Bonferroni’s multiple comparison tests (Fig. S2a,b).

Pups’ retrieval latency and duration were measured on PPD1, PPD2, and PPD3. The mean pups’ retrieval latencies were 27.7 ± 3.4 s, 18.4 ± 2.6 s, 20.7 ± 4.8 s on PPD1, PPD2, PPD3 respectively for control mice, and 30.1 ± 5.7, 19.9 ± 3, and 16.2 ± 3 on PPD1, PPD2, PPD3 respectively for OXTR-cKO. The mean retrieval durations and latencies were 78.9 ± 9, 63.7 ± 9.9, 71.8 ± 17.9 on PPD1, PPD2, PPD3 respectively for control mice, and 97 ± 14.7, 59.9 ± 11, 52.7 ± 8.1 on PPD1, PPD2, PPD3 respectively for OXTR-cKO. Two-way ANOVA revealed no difference in the retrieval latencies or durations between the control and the OXTR-cKO mice on any days *P* > 0.05 (Fig. 2d,e).

Maternal aggression was measured on PPD7 and the average numbers of attacks on the stranger male mouse were not significantly different between the two groups; 17.22 ± 3.73 and 13.42 ± 4.46 by OXTR-KO and the control mothers respectively, *P* > 0.05 unpaired *t* test (Fig. 2f).

In virgin mice, Pups’ retrieval latency and duration were measured in four consecutive days. Two-way ANOVA revealed no difference in the retrieval latencies or durations between the control and the OXTR-cKO mice on any days *P* > 0.05 (Fig. 2g,h). It is evident that the latencies and durations of pups’ retrieval by mothers are much lower than in virgin mice.

### Deletion of the OXTR from MCH neurons produces diverse effects on depressive behavior in sexually naïve and postpartum female mice

**Figure 3:**
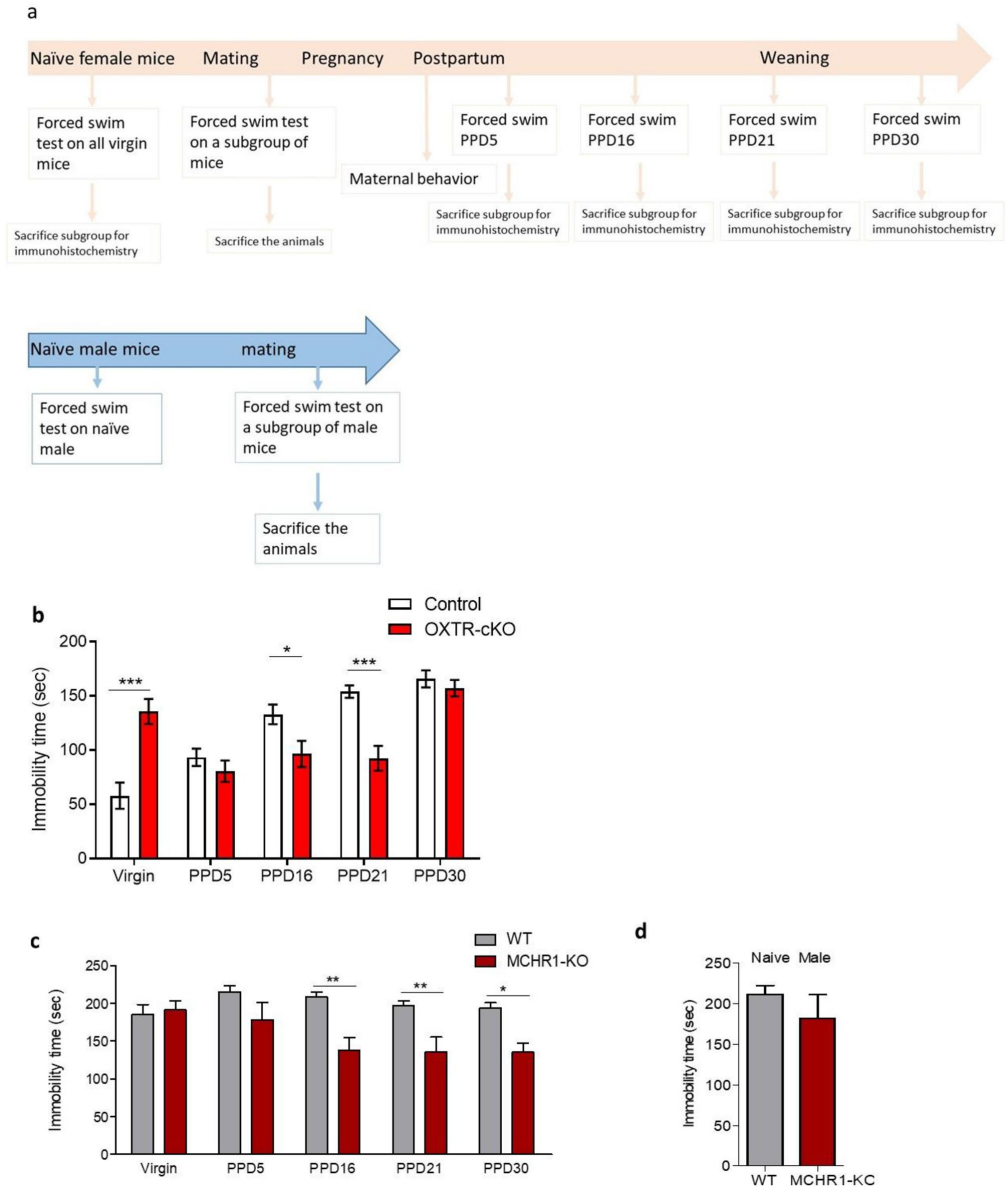
OXTR deletion from MCH neurons produces discrete effects on depressive behavior. (**a**) Schematic diagram of the behavioral experimental design. (**b**) Immobility times in control and OXTR-cKO female mice: two-way ANOVA analysis of immobility time revealed the following changes in OXTR-cKO mice: increase in virgin females, no change on PPD5, decrease on PPD16 and PPD21, and no change on PPD30 (Virgin: n_control_ = 19, n_OXTR-cKO_ = 19, ***P < 0.001; PPD5: n_control_ = 24, n_OXTR-cKO_ = 24, *P* > 0.05; PPD16: n_control_ = 24, n_OXTR-cKO_ = 24, *P < 0.05; PPD21: n_control_ = 24, n_OXTR-cKO_ = 24, ***P < 0.001, PPD30: n_control_ = 10, n_OXTR-cKO_ = 10, *P* > 0.05, Bonferroni post-test). (**c**) Immobility time in female WT and MCHR1-KO mice: immobility time in MCHR1-KO is not changed in virgin females and in PPD5, and is decreased in PPD16, PPD21 and PPD30 mice (Virgin: n_WT_ = 7, n_MCHR1-KO_ = 7, *P* > 0.05; PPD5: n_WT_ = 6, n_MCHR1-KO_ = 6, *P* > 0.05; PPD16: n_WT_ = 8, n_MCHR1-KO_ = 6, **P < 0.01; PPD21: n_WT_ = 7, n_MCHR1-KO_ = 6, **P < 0.01, PPD30: n_WT_ = 6, n_MCHR1-KO_ = 6, *P < 0.05, Bonferroni post-test). (**d**) Immobility times in naive control and OXTR-cKO male (n_WT_ = 6, n_MCHR1-KO_ = 6, *P* > 0.05, unpaired *t* test).

We examined the effect of OXTR deletion from MCH neurons on emotional behavior and whether maternal experience modifies these effects (Fig. 3a). Virgin OXTR-cKO female mice displayed a two-fold increase in immobility time, *P* < 0.001, two-way ANOVA indicating an increase in depressive behavior. However, on PPD5, control and OXTR-cKO displayed comparable immobility times, *P* > 0.05, two-way ANOVA, followed by Bonferroni post-test (Fig. 3b). Most interestingly, the deletion of OXTR from MCH neurons produced significant reductions in immobility times on PPD16 and PPD21 compared to the control group on the same PPDs, *P* < 0.05 and < 0.001 for PPD16 and PPD21 respectively, two-way ANOVA, followed by Bonferroni post-test. OXTR-cKO mice and their control littermates exhibited similar levels of immobility on PPD30 (9 days after weaning), *P* > 0.05, two-way ANOVA.

### MCHR1-KO exhibit decreased postpartum depression-like behavior

We examined immobility times of the forced swim test in sexually naïve male and female MCHR1-KO and their littermate WT mice (n = 6/condition). We also measured immobility times in female MCHR1-KO and their littermate WT mice during early and late postpartum stages. Naïve male and female MCHR1-KO mice did not display differences in immobility time compared to their matching WT mice (male mice: *P* > 0.05 *t* test; female: *P* > 0.05 two-way ANOVA followed by Bonferroni post-test, Fig. 3c,d). However, MCHR1-KO postpartum females exhibited reduced immobility time compared to WT on PPD16, PPD21, and PPD30 but not PPD5 (*P* > 0.05, *P* < 0.01, *P* < 0.01, and *P* < 0.05 on PPD5, PPD16, PPD21, and PPD30 respectively, two-way ANOVA followed by Bonferroni post-test, Fig. 3c,d).

### Maternal experience and OXTR deletion from MCH neurons cause remapping of brain Arc expression

Using immunostaining of Arc, we tested whether OXTR deletion from MCH neurons is associated with changes in the neuronal activity (determined by measuring the number of Arc^+^ cells) in the hypothalamic nuclei that contain MCH (LH) and oxytocin (PVN and SON).

**Figure 4:**
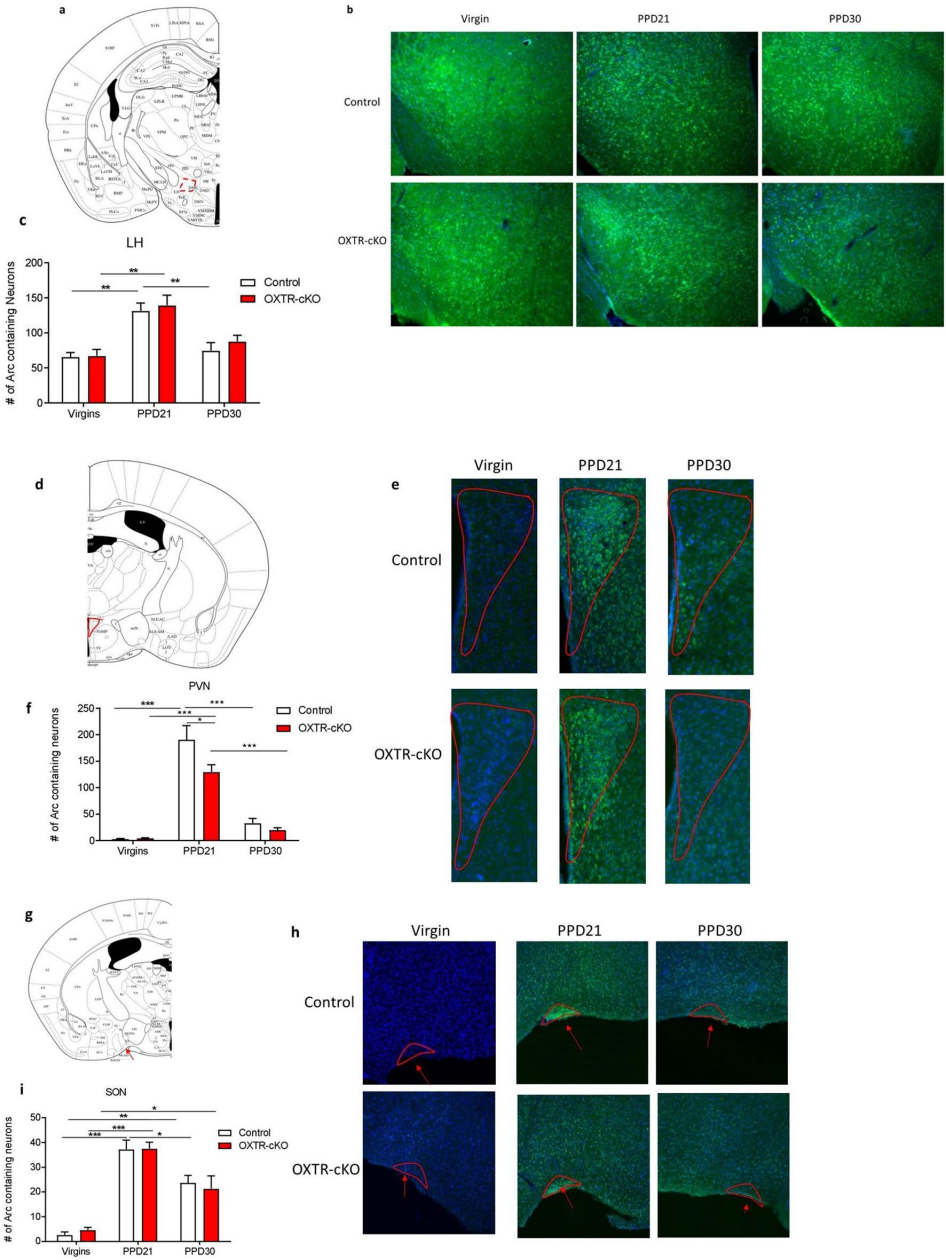
Maternal experience and OXTR deletion from MCH neurons cause alterations of Arc expression in the LH, PVN, and SON. (**a**) Location of the Arc immunostaining in the LH on mouse brain section (Image Credit: The Mouse Brain Atlas^36^). (**b**) Representative image of Arc immunostaining in the LH in the different groups. Scale: 20 µm. (**c**) Number of Arc positive neurons in the LH (virgin: n_control_ = 6, n_OXTR-cKO_ = 5; PPD21: n_control_ = 7, n_OXTR-cKO_ = 6; PPD30: n_control_ = 7, n_OXTR-cKO_ = 4, **P < 0.01, two-way ANOVA followed by Tukey's multiple comparisons test). (**d**) Location of the Arc immunostaining in the PVN on mouse brain section (Image Credit: The Mouse Brain Atlas^36^). (**e**) representative image of Arc immunostaining in the PVN in the different groups. Scale: 20 µm. (**f**) number of Arc positive neurons in the PVN (virgin: n_control_ = 7, n_OXTR-cKO_ = 7; PPD21: n_control_ = 7, n_OXTR-cKO_ = 7; PPD30: n_control_ = 7, n_OXTR-cKO_ = 5, *P < 0.05, ***P < 0.001, two-way ANOVA followed by Tukey's multiple comparisons test). (**g**) Location of the Arc immunostaining in the SON on mouse brain section (Image Credit: The Mouse Brain Atlas^36^). (**h**) Representative image of Arc immunostaining in the SON in the different groups. Scale: 20 µm. (**i**) Number of Arc positive neurons in the SON (virgin: n_control_ = 6, n_OXTR-cKO_ = 5; PPD21: n_control_ = 5, n_OXTR-cKO_ = 4; PPD30: n_control_ = 6, n_OXTR-cKO_ = 6, *P < 0.05, **P < 0.01, ***P < 0.001, two-way ANOVA followed by Tukey's multiple comparisons test).

In the three nuclei both control and OXTR-cKO females exhibited on PPD21 significantly higher numbers of Arc positive neurons than in virgin females, and on PPD30 the levels of Arc expression returned to the virgin levels (Fig. 4a–i). The deletion of OXTR from the MCH neurons did not cause any change in the number of Arc positive neurons in the LH, SON in any group, *P* > 0.05 (Fig. 4a–f). However, OXTR-cKO mice exhibited reduced number of Arc positive neurons in the PVN on PPD21 only (Fig. 4g–i).

**Figure 5:**
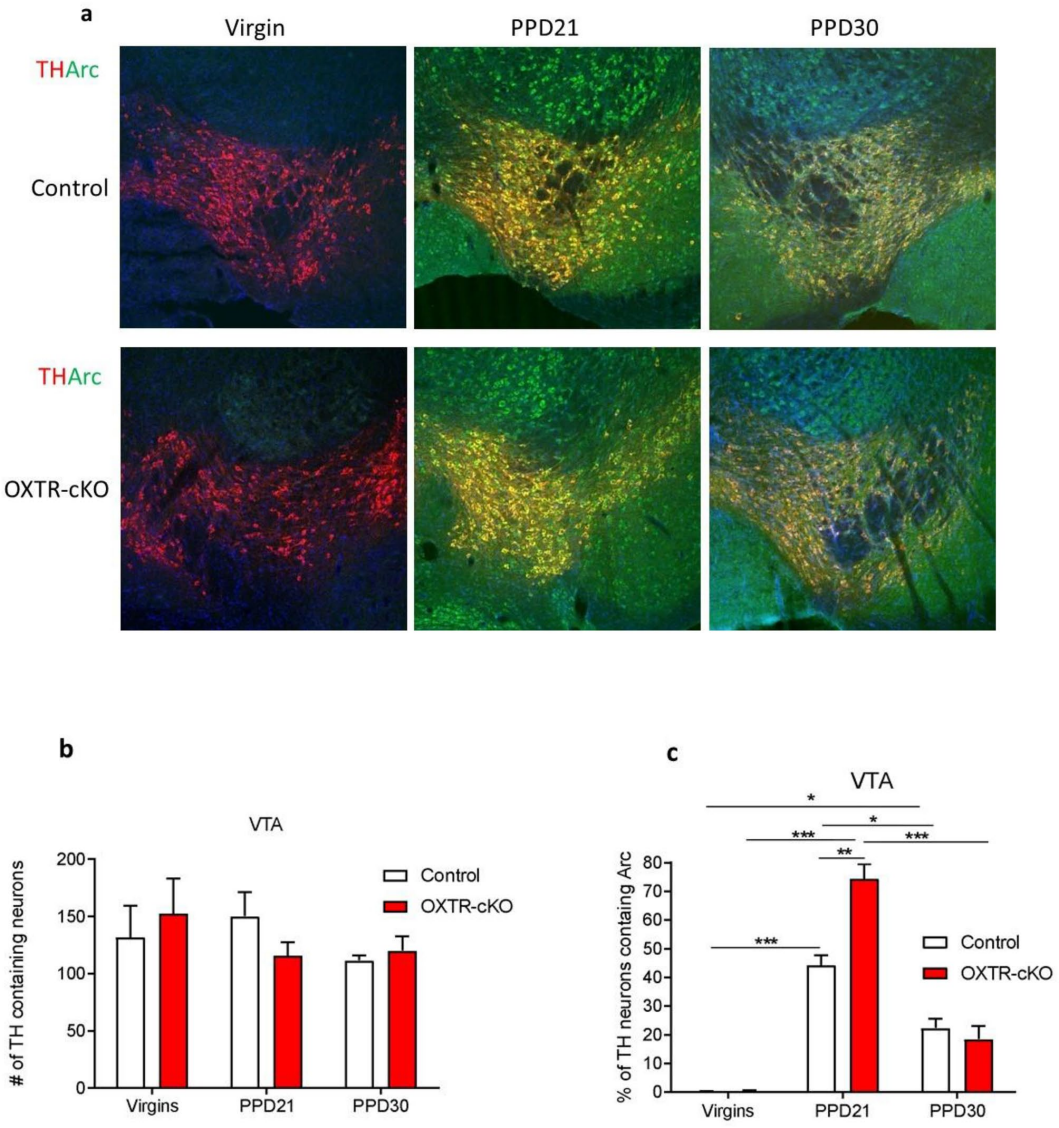
Maternal experience and OXTR deletion from MCH neurons cause alterations of Arc expression in the VTA dopamine neurons. (**a**) Representative image of Arc and TH immunostaining in the VTA in the different groups. Scale: 20 µm. (**b**) Number of TH positive neurons in the VTA (virgin: n_control_ = 3, n_OXTR-cKO_ = 3; PPD21: n_control_ = 3, n_OXTR-cKO_ = 5; PPD30: n_control_ = 5, n_OXTR-cKO_ = 5, *P* > 0.05, two-way ANOVA followed by Tukey's multiple comparisons test); (**c**) percentage of TH positive neurons containing in the VTA (virgin: n_control_ = 3, n_OXTR-cKO_ = 3; PPD21: n_control_ = 3, n_OXTR-cKO_ = 5; PPD30: n_control_ = 5, n_OXTR-cKO_ = 5, *P < 0.05, **P < 0.01, ***P < 0.001, two-way ANOVA followed by Tukey's multiple comparisons test).

Because the reward and fear systems are involved in maternal behaviors and depressive behavior, we tested whether the changes in depressive behavior are associated with changes in the neuronal activity of the VTA and amygdala, and whether parenting affects Arc expression topography. The deletion of OXTR from the MCH neurons did not alter the total number of Arc positive neurons in the VTA in virgin or mother mice (PPD21 and PPD30), *P* > 0.05 (Fig. 5a–c). However, the percentage of dopaminergic neurons (TH positive neurons) that express Arc on PPD21 was higher in OXTR-cKO mice than in the control mice (*P* < 0.01). Furthermore, the total number of Arc positive neurons were significantly higher on PPD21 in both control and OXTR-cKO mice than in virgin mice, and these levels were reduced on PPD30 to levels comparable to virgin mice (Fig. 5a–c).

**Figure 6:**
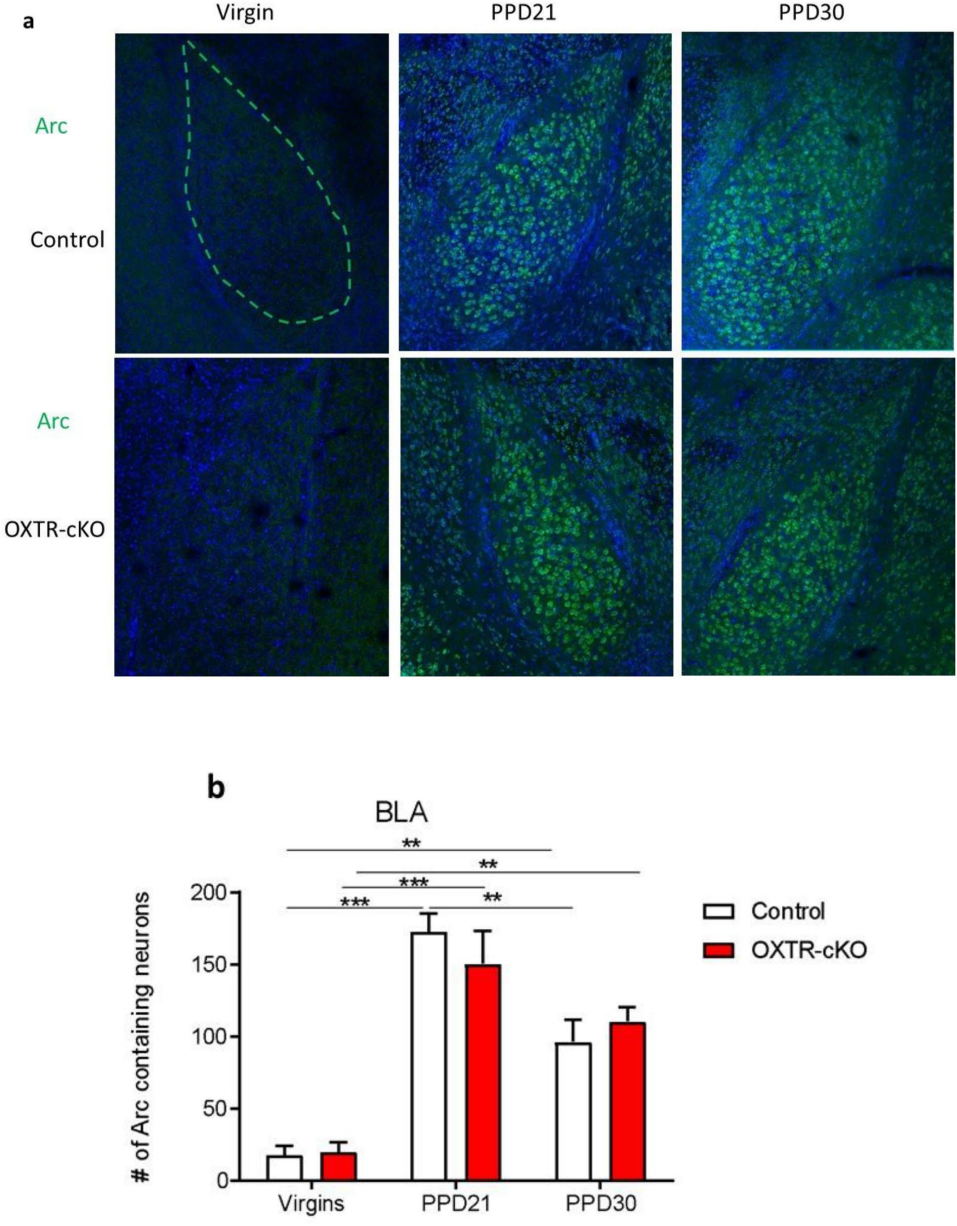
Maternal experience and OXTR deletion from MCH neurons cause alterations of Arc expression in the amygdala. (**a**) Representative image of Arc in the BLA in the different groups. Scale: 20 µm. (**b**) Number of Arc positive neurons in the BLA (virgin: n_control_ = 6, n_OXTR-cKO_ = 4; PPD21: n_control_ = 7, n_OXTR-cKO_ = 6; PPD30: n_control_ = 7, n_OXTR-cKO_ = 5, **P < 0.01, ***P < 0.001, two-way ANOVA followed by Tukey's multiple comparisons test).

The basolateral amygdala (BLA) plays a crucial role in the modulation of emotions and it is known to receive oxytocin signaling^39^. In the basolateral amygdala (BLA), the total number of Arc positive neurons were significantly higher in the PPD21 mice than in the virgin mice, and these levels returned on PPD30 to levels comparable to virgin mice (Fig. 6a,b). The deletion of OXTR from the MCH neurons did not cause any change in the total number of Arc positive neurons in the BLA neither in virgin nor postpartum mice (PPD21 and PPD30), (*P* > 0.05, Fig. 6a,b).

### Mating behavior affects mood responses to OXTR deletion from MCH neurons in a sex-dependent manner

**Figure 7:**
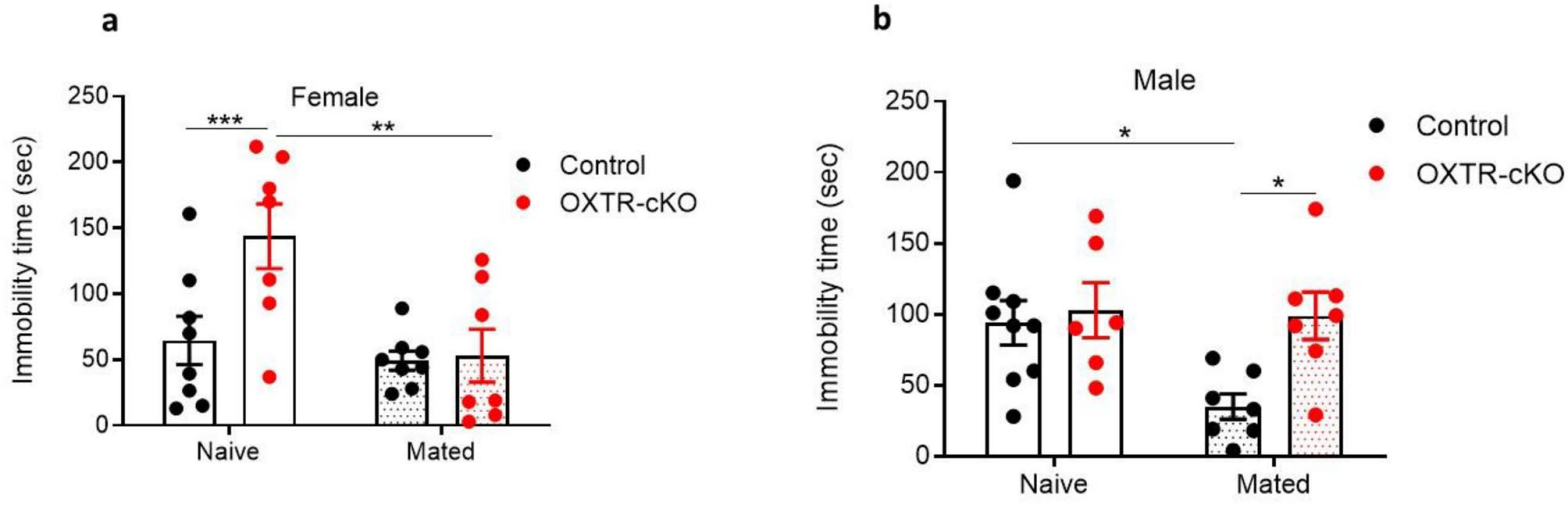
Anti-depressant effect of mating is sex-dependent and is abolished by OXTR deletion from MCH neurons. (**a**) Effect of mating on immobility times in female control and OXTR-cKO mice, n_control_ = 8, n_OXTR-cKO_ = 7, **P < 0.01, ***P < 0.001, two-way ANOVA analysis, followed by Tukey's multiple comparisons test. (**b**) Effect of mating on immobility times in male control and OXTR-cKO mice, naive: n_control_ = 7, n_OXTR-cKO_ = 7, *P < 0.05, two-way ANOVA analysis, followed by Tukey's multiple comparisons test.

To examine the effects of mating on emotional behavior, control and OXTR-cKO male and female mice were allowed to mate (within the same genotype group) for 3 days, and were tested 24 h after the last contact in the forced swim test. Mating activity did not change the immobility time in the control female mice (*P* > 0.05 mated control mice compared to naïve control mice), suggesting that sexual activity does not affect the basal levels of mood in female mice (Fig. 7a). However, mating restored the elevated immobility time observed in OXTR-cKO mice to levels comparable to those seen in control mice (*P* > 0.05: mated OXTR-cKO female mice compared to naive control female mice, and *P* < 0.01: mated OXTR-cKO female mice compared to naïve OXTR-cKO female mice, two-way ANOVA followed by Tukey post-test, Fig. 7a). These results indicate that mating alleviates the depressive behavior evoked by OXTR deletion from MCH neurons.

In contrast to female mice, naïve control and OXTR-cKO male mice displayed similar immobility time (*P* > 0.05, Fig. 7b), indicating that OXTR deletion from the MCH neurons does not affect the basal levels of emotional behavior in male mice. Mating, however, decreased immobility time in the control group (*P* < 0.05 mated control mice compared to naïve control mice, Tukey's multiple comparisons test), but not in the OXTR-cKO group, (*P* > 0.05 mated OXTR-cKO mice compared to naïve OXTR-cKO mice, Tukey's multiple comparisons test, Fig. 7b). This finding suggests that the deletion of OXTR from the MCH neurons does not affect the basal levels of emotional behavior, but it diminishes the anti-depressant effect of mating in male mice.

## Discussion

In the present study, we uncovered the role of the oxytocin-MCH signaling pathway in mood regulation. Our work resulted in four novel findings. First, we identified a selective role for oxytocin-MCH signaling in mood regulation but not in maternal behavior. Second, we demonstrated that the effects of OXTR deletion from MCH neurons on mood is sex-dependent. Third, we showed that mating and parenting experiences shape basal mood and mood responses to OXTR deletion in MCH neurons. Finally, we showed that parenting experience and OXTR deletion from MCH neurons cause remapping of brain Arc expressions, and that lower depressive behavior is associated with higher and lower Arc expression in the VTA and PVN respectively.

Oxytocin and MCH systems share several physiological actions such as the control of maternal care, sexual behavior, and emotions^15–24^. During parturition, oxytocin’s major role is to turn on the brain maternal circuit for the initiation of maternal behavior. We report here, however, that the requirement for oxytocin increases with the progress of postpartum periods, revealed by the increased oxytocin mRNA levels on the late stage of postpartum period compared to virgin, pregnant and early postpartum stages, implicating discrete functions of oxytocin during the late postpartum stages. Interestingly, oxytocin patterns of expressions were similar to that of MCH expressions across pregnancy and postpartum periods^27^, supporting an interaction between the two systems to regulate particular physiological processes during these periods.

Despite the extensive studies implicating oxytocin signaling in different brain regions in maternal behaviors, the role of oxytocin in the lateral hypothalamus has remained unaddressed. We previously showed that pharmacological blockade of the MCH system can block specific oxytocin-mediated actions, particularly repetitive behavior^29^. We also showed that pharmacological and genetic disruptions of MCH signaling cause deficits in maternal behaviors in naïve and postpartum mice^27,30^. Therefore, it is reasonable to speculate that the two systems interact to regulate maternal behavior and emotions.

Consistent with our previous report using the OTR-Venus knock in mouse^29^, our present results, using OXTR-RNAScope in conjunction with immunofluorescent staining for MCH, revealed that about 67% of MCH neurons express OXTR-mRNA. These results also suggest that in the LH, OXTR is primarily expressed in the MCH neurons but less in other neurons. In agreement, electrophysiological studies reported that oxytocin depolarizes MCH cells but not other neurons in the LH^28^, supporting the very selective innervation and activation of MCH neurons by oxytocin.

Disruptions of oxytocin and MCH systems are well known to produce impairments in several aspects of maternal behaviors^3,14,27,30,40–45^. However, we found that the disruption of oxytocin-MCH pathway, through OXTR deletion from MCH neurons, did not affect maternal behaviors, evidenced by the normal pups’ retrieval, nest building, and maternal aggression in the OXTR-cKO mice. These findings indicate that even though MCH and oxytocin systems share the control of maternal behaviors, oxytocin-MCH signaling is not particularly involved in regulating these behaviors. The results also suggest that alternate MCH and oxytocin pathways, separately regulating maternal behaviors, are likely intact in OXTR-cKO mice.

The most intriguing finding is that oxytocin-MCH signaling controls mood, and that this action is sexually dimorphic and experience-dependent (maternal and mating experiences). OXTR deletion from MCH neurons did not affect mood in naïve male; however, it resulted in diverse changes in mood in female mice in naïve vs across postpartum stages. Thus, OXTR-cKO mice exhibited an increase in immobility time in virgin female, a return to control levels on PPD5 (early postpartum stage), a decrease in PPD16(early postpartum stage) and PPD21 (weaning day), and a return to control levels on PPD30 (single-housed mothers for 9 days post-weaning). These findings provide, for the first time, evidence for an essential role of maternal experience in modifying the emotional responses mediated by OXTR-MCH circuit. In line with these results, we found that the germline deletion of MCHR1 produces mood patterns relatively similar to those seen in OXTR-cKO mice. MCHR1 germline deletion did not affect mood in early postpartum female mice(PPD5), however, it caused a significant reduction in depressive behavior on PPD16, PPD21, and PPD30. The only discrepancies in the emotional responses between MCHR1-KO and OXR-cKO are seen in naïve female mice (increased immobility time in OXTR-cKO and no change in MCHR1-KO mice), and on PPD30 (no change in OXTR-cKO mice while reduced immobility time in MCHR1-KO). Two hypotheses are proposed to explain these discrepancies. First, given the genetic background differences between the two mouse strains that were used in the current study, and noting that the WT background for MCHR1-KO mice, Taconic mice, display considerably higher immobility time than the control mice of OXTR-cKO, reaching in some animals the cutoff time. Therefore, it is possible that an increase in immobility time in these animals could not be detected in our experimental setting. Second, in response to the OXTR-deletion, MCH system may undergo slow but long-term neuroplasticity, which persists for several days after the removal of maternal stimuli.

The immense changes of emotional states in females as they become mothers are associated with structural and functional plasticity of the mothers’ brains including morphological and physiological plasticity in the PVN and SON of the hypothalamus^46,47^. Since oxytocin orchestrates the mother’s adaptations during postpartum period, we examined the neuroplasticity associated with the maternal experience by measuring the expression of activity-regulated cytoskeletal gene (Arc). Arc is an immediate early gene that plays core roles in experience-dependent synaptic strength and neural plasticity in frontal cortex and other brain regions involved in cognitive functions and emotions^48–53^.

We report a massive increase in Arc expression during late postpartum stage in various brain regions including the LH, PVN, SON, VTA, and BLA, in both the control and OXTR-cKO female mice. This is the first report on the remapping of Arc expression by maternal experience. Our results suggest that Arc expression can serve as a marker for mapping the neural substrates recruited by female brain to induce neuroplasticity and prepare females for parenthood. Interestingly, OXTR deletion from the MCH neurons caused opposite alterations in Arc expression on PPD21 in the PVN (decrease) and VTA (increase).

Noticeably, Arc expression was associated with depressive behavior in female mice. Thus, increased depressive behavior was associated with increased Arc expression in the reward circuit (VTA), while the decreased depressive behavior was associated with decreased Arc expression in the stress circuit (PVN). The oxytocin-MCH signaling likely facilitates a reciprocal stimulation of PVN-oxytocin neurons and LH-MCH, and it interacts with the reward and fear circuits to discretely regulate mood in virgin and postpartum stages of the life of female mice.

Finally, we found that mating experience modifies the emotional responses induced by OXTR deletion from MCH neurons. Oxytocin is a crucial regulator of sexual function^54–58^, though it is still unclear whether oxytocin evokes sexual arousal or is a byproduct of it. Sexual activity and mating behavior are known to produce relaxation, calmness, and sedation in humans and animals^9,59–61^. The release of oxytocin from the PVN has been shown to mediate these antidepressant and anxiolytic effects of sexual activity and mating behavior^9,10^.

We show that the effects of mating on depressive behavior is sex-dependent and that it is mediated through the activation of the oxytocin-MCH pathway. Mating activity reduced depressive behavior in normal male mice, and the deletion of OXTR from MCH neurons in male mice blocked the anti-depressant effects of mating activity, indicating that the oxytocin-MCH pathway mediates the anti-depressant effects of mating in males. Mating activity did not affect depressive behavior in normal females, however, it reversed the evoked depressive behavior induced by OXTR deletion from MCH neurons. These results suggest that the activation of oxytocin-MCH pathway is important for the regulation of the basal levels of mood in female mice, and that mating alleviates evoked- but not basal depression.

In conclusion, we illustrate the role of a previously undefined hypothalamic circuit in regulating mood. We prove that the mood-modulating effects of oxytocin-MCH signaling are sex-dependent, and associated with alterations in Arc expression in reward and stress systems, and that mating and parental experiences shape these actions of oxytocin-MCH. These data suggest that oxytocin-MCH pathway can serve as a potential therapeutic target for major depressive disorder and postpartum mood abnormalities. Finally, we uncover the discrete effects of sexual activity on mood in male and female animals. We prove that while sexual activity reduces basal levels of depression in male mice, it can only reduce the evoked depression in female mice.

## Supplementary information

Supplementary Figures.

## Data Availability

All data generated or analyzed during this study are included in this published article (and its Supplementary Information files).
