# Supplementary material for: Mating and parenting experiences sculpture mood-modulating effects of oxytocin-MCH signaling
Source: Sci Rep. 2020 Aug 12;10:13611. doi: 10.1038/s41598-020-70667-x (PMC7423941; doi:10.1038/s41598-020-70667-x)
Supplement: Supplementary file 1 — Supplementary Figures. [file 41598_2020_70667_MOESM1_ESM.docx]

**Phan et al, supplementary material**

**Mating and Parenting Experiences Sculpture Mood-Modulating Effects of**

**Oxytocin- MCH Signaling**

Joseph Phan^1a^, Lamees Alhassen^1a^, Allan Argelagos^1^, Wedad Alhassen^1^, Benjamin Vachirakorntong^1^, Zitong Lin^1^, Nayna Sanathara^1^, Amal Alachkar^1,2^*

^1^ Departments of Pharmaceutical Sciences, University of California Irvine, CA 92697

^2^ Institute for Genomics and Bioinformatics, School of Information and Computer Sciences, University of California-Irvine, CA 92697

**^a^ These authors contributed equally to the work**

*** Corresponding Author**:

AmalAlachkar

Department of Pharmaceutical Sciences

University of California, Irvine

356A Med Surge II

Irvine CA, 92697-4625

Phone; 949-824-2522

[aalachka@uci.edu](mailto:aalachka@uci.edu)

**Figure. S1**. **OXTR-cKO on MCH neurons manifest no significant difference in comparison to the control group with regards to initial litter size, cannibalism, or pups’ survival**. a. Initial litter size for control and OXTR-cKO mothers displayed no significant difference when using two-tailed unpaired t-test (t=0.07, p=0.42). b. Pups’ survival rate of control and OXTR-cKO showed no significant difference across five postpartum days when using two-way ANOVA; c Cannibalism of OXTR-cKO and control mothers showed no significant difference when using two-tailed unpaired t-test (t=0.28, p=0.86). (*P* > 0.05), d. nest building scores showed no significant difference across three days postpartum, *P* > 0.05, two-way ANOVA.


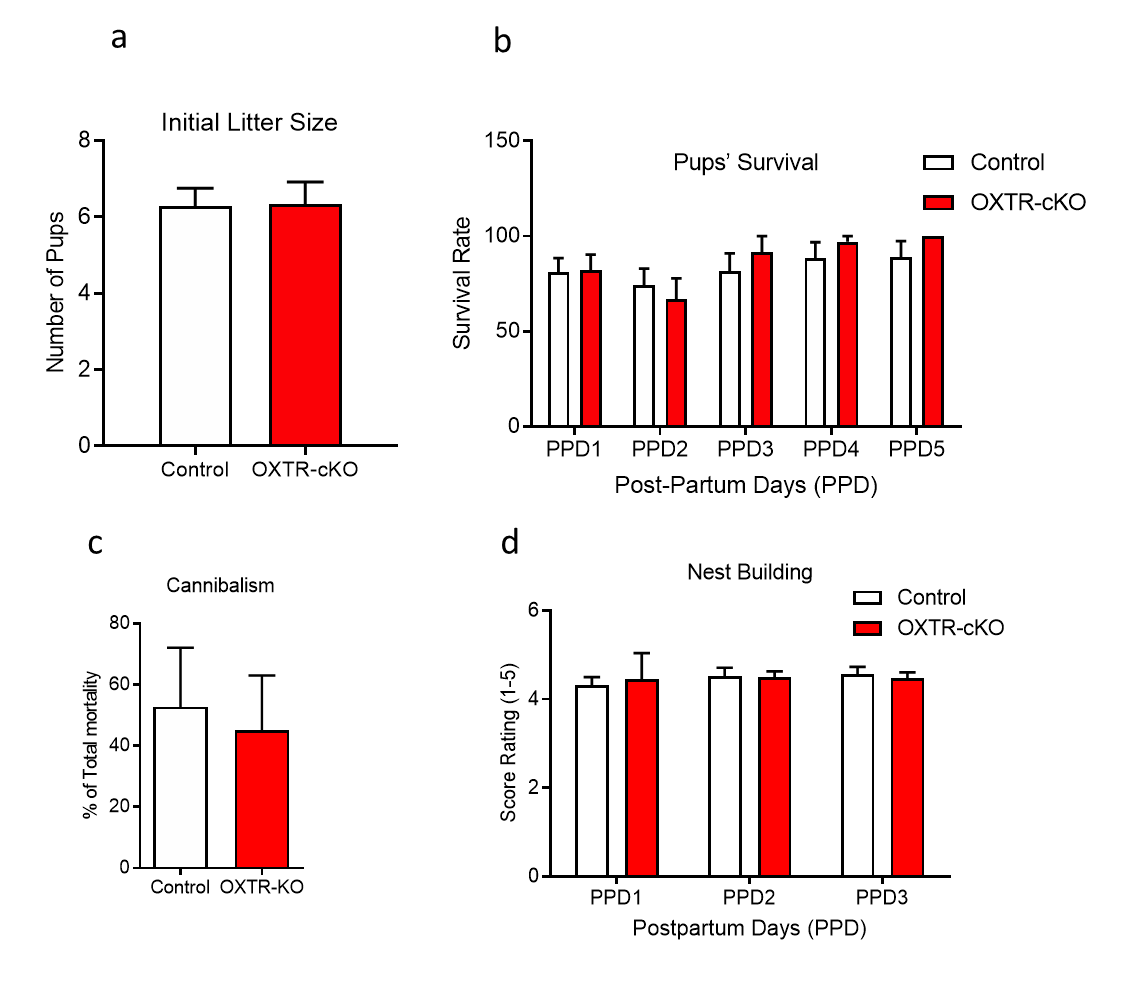


**Figure. S2**. **Oxytocin effect on MCH neurons has no effect on mother’s milk production, which causes no significant difference in pups’ weight**. a. Pups’ weight in grams over twenty-one postpartum days, in which the control and OXTR-cKO show no significant difference (adjusted p value > 0.99 over twenty-one days). b. Mother’s milk production measured as g/day/pup until pups’ were twenty days old. No significant differences were seen when comparing the control against the OXTR-cKO (adjusted p value > 0.99 over twenty-one days).

a b
